# 

**Published:** 1849-09

**Authors:** 


### CONTENTS

### OF NO. III.

---

### ORIGINAL COMMUNICATIONS.

### ESSAYS AND CASES.

ART. I.—Spiritus Pyroxylicus in the treatment of Diarrhea and Dysentery. By DAVID W. YANDELL, M. D., of Louisville, Ky. - - - - 185

ART. II.—Report of a Case of Hydrophobia, which occurred in Louisville, Ky. By R. C. HEWETT, M. D. 202

ART. III.—Report of Cases. By B. I. RAPHAEL, M. D., Attending Surgeon to the Louisville Marine Hospital, 212

### REVIEWS.

ART. IV.—Manual of Physiology. By WILLIAM SENHOUSE KIRKES, M. D. Assisted by JAMES PAGET, Lecturer on General Anatomy and Physiology, at St. Bartholomew's Hospital, with 118 illustrations on wood, - 221

ART. V.—Human Anatomy. By JONES QUAIN, M. D. Edited by RICHARD QUAIN, F. R. S., and WILLIAM SHARPEY, M. D., F. R. S., Professors of Anatomy and Physiology in University College, London, - 233

### SELECTIONS FROM AMERICAN AND FOREIGN JOURNALS.

Zymotic Diseases—Typhus and Typhoid Fevers, Diagnosis of 245

Photophobia, resulting from exalted sensibility of the sensitive branch of the fifth pair going to the eye from irritation of the sensitive branch of the same nerve going to the teeth, - - - - - - 247
Nurse's Sore Mouth, - - - - - - 251
Aphtha, - - - - - - - 251
On the Employment of Nitrate of Silver as a Vesicant, - 252
The Mechanical Leech of MM. Alexandre & Co. of Paris, - 254
On the Analogy and Differences between Tubercle and Scrofula, 255
On the Simultaneous Development of Variola and Vaccinia, - 256
Arterial Compression as an Antiphlogistic, - - 259
Femoral Aneurism, - - - - - - 260

ORIGINAL INTELLIGENCE.

Cholera in Rutherford county, Tenn. - - - 269
Cholera as Influenced by Locality, - - - 271
Cholera and Electricity, - - - - - 273
Clinical Teaching—the New Amphitheater at the hospital, 274
Proceedings of the American Association for the advancement of Science, - - - - - 275

